# Comparison of two culture methods for the enumeration of *Legionella pneumophila* from potable water samples

**DOI:** 10.2166/wh.2021.051

**Published:** 2021-06

**Authors:** Laura A. Boczek, Min Tang, Casey Formal, Darren Lytle, Hodon Ryu

**Affiliations:** U.S. Environmental Protection Agency, Office of Research and Development, Center for Environmental Solutions and Emergency Response, 26 West Martin Luther King Drive, Cincinnati, OH 45268, USA

**Keywords:** BCYE agar, Legiolert, *Legionella pneumophila*, potable water, simulated home plumbing system

## Abstract

Legionella infections have steadily increased in the United States over the last 20 years, and most of these infections have been attributed to contaminated water. The gold standard for confirmation of *Legionella* presence in water is culturing with Buffered Charcoal Yeast Extract (BCYE) agar. Following many modifications, this method is still time-consuming, expensive, and can take longer than 10 days for full confirmation. The Legiolert is a newer and simpler culture product that is claimed to be able to quantify *Legionella pneumophila* in 7 days with high sensitivity and specificity and does not need further confirmation for the presence of *L. pneumophila*. This study compared the culturability of *L. pneumophila* occurring in a simulated home plumbing system using both Legiolert and BCYE agar methods. Out of 185 water samples, Legiolert and BCYE method detected *L. pneumophila* in 83 and 85% of the samples, respectively. The two methods were determined to be statistically equivalent for culturability of *L. pneumophila*, though the detected levels by Legiolert were slightly higher than the BCYE method. The molecular confirmation of positive (*n* = 254) and negative wells (*n* = 82) with Legiolert also showed a high specificity of 96.5% (i.e., 3.5% false positives (9/254) and 0% false negatives (0/82)).

## INTRODUCTION

Legionnaires’ disease is a respiratory disease that causes a severe form of pneumonia. It was first discovered in 1976 when over 200 individuals attending an American Legion Conference contracted the bacterium *Legionella pneumophila* from the hotel’s cooling tower air conditioning system (Brenner *et al.* 1978, 1979). There are more than 58 different species of *Legionella*, and over 25 of these species have been implicated in human disease (McDade *et al.* 1977). *Legionella* spp. are gram negative, motile, rod-shaped bacteria that are known to live in aquatic environments. *L. pneumophila* is the species that is responsible for 90% of Legionaries’ disease, and within the species *pneumophila*, there are 15 different serogroups. *L. pneumophila* serogroup 1 is responsible for 85% of the infections caused by *L. pneumophila* (Yu *et al.* 2002). Disease burden of Legionnaires’ Disease has increased steadily over the last 20 years in the United States (Diederen 2008; Hicks *et al.* 2011; Demirjian *et al.* 2015). The Centers for Disease Control and Prevention (CDC) estimates that there were about 1,000 cases of Legionnaires’ disease in the year 2000, and the estimate for 2018 was over 9,000 infections (CDC 2011, 2015). It is more than likely due to a combination of things including aging populations, climate change leading to warmer water which favors *Legionella*, and energy conservation in buildings leading to increased water age, increased nutrient load, and lower disinfectant residuals. *Legionella* spp. is unique in that it can grow in a wide span of water temperatures ranging from 25 to 45 ^◦^C. Therefore, the water environment within water distribution systems, premise plumbing systems in buildings (e.g., hospitals, schools, and homes), cooling towers, and other water facilities are usually conducive for growth and proliferation of *Legionella* spp. Specifically, hot water systems in premise plumbing are complex and typically have a low or zero disinfectant residual. Many property owners operate these hot water systems at temperatures that are ideal for growth of *Legionella* to avoid scalding concerns. Facilities such as hospitals where immunocompromised populations are present are particularly sensitive to *Legionella* risks.

*Legionella* spp. can be difficult to culture out of water systems and to recover from environmental sources. Buffered Charcoal Yeast Extract (BCYE) Agar has been used historically as the culture media for growth of *Legionella*. However, *Legionella* is a slow-growing bacterium. It takes 7–10 days for incubation, and then, it needs further testing for verification of genus, species, as well as serogroups using latex agglutination tests specific for *L. pneumophila*. BCYE media can also be overgrown by other microorganisms present in the water sample. This is a common occurrence due to the slow-growing nature of *Legionella* spp. compared with other organisms that can grow faster. If overgrowth by non-target organism is a concern, treatment techniques such as acid treatment, heat treatment, and the use of selective additives (e.g., antibiotics) need to be added to the sample or media (U.S. Department of Health and Human Services 2005). These treatments will suppress the growth of the non-target organisms significantly, but they lead to a lower estimate of *Legionella* spp. in the samples due to lower culturability after treatment (Ta *et al.* 1995; Leoni & Legnani 2001; Bartie *et al.* 2003). Therefore, there is a need for a robust, easy method that gives reliable results on the presence of these organisms in water systems. In situations where *Legionella* concentrations are low, it is important to capture these organisms for an accurate assessment of the water source. Moreover, the availability of better and easier detection methods for these bacteria is crucial to aid water system and facility managers in monitoring microbial water quality in water distribution systems and implementing appropriate building water management plans.

IDEXX Laboratories Inc. (Westbrook, ME) has created the Legiolert product which can grow and detect the presence of *L. pneumophila* from potable and non-potable water samples in 7 days. Legiolert uses the Quanti-Tray system (IDEXX Laboratories Inc.) which allows for enumeration of *L. pneumophila*, and the trays can be investigated to pull out an individual isolate for further testing. The manufacturer claims that there is no need for further verification for *L. pneumophila* due to the high selectivity against non-pneumophila species. More recently, research groups from Europe and the United States have reported that Legiolert yielded a higher sensitivity (i.e., higher counts of correctly identified positive samples) and had a higher specificity (i.e., higher counts of correctly identified negative samples) for *L. pneumophila* compared with the conventional BCYE agar method (Sartory *et al.* 2017; Petrisek & Hall 2018; Spies *et al.* 2018; Inoue *et al.* 2020; Scaturro *et al.* 2020). While this method shows promising practical applications (i.e., simplistic and less laborious) over the traditional culture method, further testing against various types of environmental water samples is necessary to ensure its reliability. The objective of this research effort was to compare qualitative and quantitative results of *L. pneumophila* from the traditional culture isolation method using BCYE agar with the Legiolert product in drinking water collected from a building model home plumbing system (HPS). In addition, the specificity of Legiolert against *L. pneumophila* in testing environmental water was investigated using a molecular confirmatory assay.

## MATERIALS AND METHODS

### Premise plumbing model system

A physical model simulated HPS was built in 2012 for conducting lead and copper corrosion studies. This simulated HPS was shown to be colonized with *Legionella* (Lytle *et al.* 2014; Lu *et al.* 2017) and thus was used as the source of the *Legionella*-containing drinking water tested using both the agar culture method and the Legiolert method. The HPS consists of an electric hot water tank, a shower head, a toilet, and four faucets that are connected via copper piping, brass fixtures, and solder joints (Supplementary Material, Figure S1). The cold water lines and the hot water tank are fed with water that is supplied by the building water supply; a water chemistry of pH 7.7 ± 0.3, alkalinity 76 ± 14 mg CaCO_3_/L, dissolved inorganic carbon 19 ± 4 mg C/L, calcium 28 ± 9 mg Ca/L, magnesium 8.4 ± 3.1 mg Mg/L, nitrate 0.88 ± 0.22 mg N/L, total phosphate 0.72 ± 0.57 mg PO_4_/L, and sulfate 58 ± 22 mg SO_4_/L during the study duration. Faucets 1, 2, and 4 had a point-of-use (POU) filtration system installed and all samples were collected in the bypass mode and only hot water samples were taken from these locations. Faucet 3 did not have a POU and, as such, both hot and cold water samples were taken at this location. The length of plumbing and water usage patterns were designed to represent a household of four persons. Water from this HPS was collected on a weekly basis from March to December 2017. Briefly, after at least 6 h of stagnation of the model home, 1 L first draw and 1 L flushed samples (second draw) were collected at a flow rate of <1 L/min using sterile polypropylene bottles containing 10% w/v of sterile sodium thiosulfate. Samples were assayed immediately for the presence of *Legionella* and heterotrophic plate count (HPC). In addition to the 1 L first draw and 1 L second draw samples, a 25 mL sample was collected each time for total and free chlorine residual, using the DPD method and a HACH pocket colorimeter.

### Legionella culture

Samples were processed for the presence of *Legionella* using the Legiolert method as well as the standard plate count method that is outlined in the CDC document entitled ‘Procedures for the Recovery of *Legionella* from the Environment’ (U.S. Department of Health and Human Services 2005). Samples analyzed for *Legionella* culture using Legiolert were processed using the method outlined by IDEXX Laboratories Inc. for potable water using the 10 mL sample volume. Briefly, 10 mL of the water sample was aseptically transferred to 90 mL of sterile Butterfields Buffer. A blister pack of Legiolert was then aseptically added to the 100 mL of buffer/sample water and shaken to dissolve the contents of the Legiolert media. Once the media was dissolved, the contents were poured aseptically into a Legiolert tray, and then sealed using a Quanti-Tray Sealer PLUS (IDEXX Laboratories Inc., Westbrook, ME). Samples were incubated according to the manufacturer for 7 days in a water-jacketed incubator with water in a large beaker for adequate humidity during the incubation at 39 °C for 7 days. The Legiolert tray has 6 big wells and 90 small wells. Wells were counted as *L. pneumophila* positive if they were turbid or brown colored after 7 days of incubation. Enumeration was determined by using IDEXX’s most probable number (MPN) table. It should be noted that standard MPN procedures use a minimum of 3 dilutions and 3, 5, or 10 replicates per dilution. In the Legiolert method, 100 mL rather than serial dilutions of samples was used, and the Legiolert MPN table is built based on the exact volume of 6 big wells and 90 small wells in the tray.

*Legionella* culture using BCYE agar was performed by filtering the collected water samples using a 0.2 μm polycarbonate filter (Sartorius, Goettingen, Germany), then transferring that membrane to a sterile 15 mL conical centrifuge tube containing 10 mL of sterile Butterfields Buffer. The tube was vortexed on high for 1 min, and then 100 μL were plated onto BCYE agar plates containing glycine, vancomycin, polymyxin B, and cycloheximide (GVPC) (Ramel, Lenexa, KS) using the spread plate method. The equivalent sample volume analyzed was approximately 10 mL (i.e., 10 mL = 1,000 mL filtered * (100 μL plated/10 mL resuspended in buffer)) which is the same volume as Legiolert. Plates were incubated for 3–7 days at 35 ^◦^C. Plates were observed for growth of *Legionella*, and then suspect colonies were transferred to a BCYE agar biplate that contained L-cystine on one half and no L-cystine on the other half.

Only cultures that grew in the presence of L-cystine were classified as presumptive *Legionella* spp. The determination of species and serogroup was determined using *Legionella* dryspot tests (Oxoid, Basingstoke, UK). Colonies confirmed as *Legionella* and serotyped were counted using a stereomicroscope and reported as colony-forming unit (CFU)/100 mL of water.

### Molecular confirmatory assay

Legiolert samples were further screened using *L. pneumophila* specific quantitative Polymerase Chain Reaction (qPCR) on randomly selected wells from the Legiolert trays. A total of 254 and 84 liquid samples collected from positive and negative wells across 14 trays were analyzed for *L. pneumophila*, respectively. The liquid samples were used directly for qPCR assay without DNA extraction and further purification. In order to mitigate PCR inhibition, 10- and 100-fold dilutions of all the samples were used, and the relative quantification of the gene copies were performed. The qPCR assay was performed in 25-μL reaction mixtures containing 1X TaqMan universal PCR master mix with AmpErase uracil-*N*-glycosylase (Applied Biosystems, Foster City, CA), 0.2 μM (final concentration) of each primer, and a 6-FAM (6-carboxyfluorescein)-labeled hydrolysis probe. The forward and reverse primers (LpneuF1, 5′-CGGAATTACTGGGCGTAAAGG-3′ and LpneuR1, 5′-GAGTCAACCAGTATTATCTGACCG-3′) and hydrolysis probe (LpneuP1, 5′-FAM-AAGCCCAGGAATTT CACAGAT-TAMRA-3′) were designed for the detection of *L. pneumophila* targeting 16S rRNA gene (Donohue *et al.* 2014). Approximate product size is 100 basepair (bp). The amplification protocol involved an initial incubation at 50 ^◦^C for 2 min to activate uracil-*N*-glycosylase, followed by 10 min of incubation at 95 ^◦^C to activate AmpliTaq Gold enzyme, and then 40 cycles of 95 ^◦^C for 15 s and 60 ^◦^C for 1 min. The qPCR assays were performed using a QuantStudio 6 Flex system (Applied Biosystems, Foster City, CA, USA). Two-independent standard curves for each qPCR assay were generated by plotting threshold cycle (CT) values against the number of target copies corresponding to serially diluted gBlock standards (IDT, Coralville, IA, USA). The target copy numbers (T) are estimated by the following equation (Ryu *et al.* 2013):

$$T=\left[\frac{D}{\left(\text{PL}\times 660\right)}\right]\times 6.022\times 10^{23}$$

where *D* (g/μL) is gBlock concentration, and PL (bp) is gBlock length in base pairs. Two no-template controls per PCR plate were used to check for cross-contamination.

### Heterotrophic bacteria culture

HPC analysis for estimating live heterotrophic bacteria was also performed on each sample using R2A agar (BD Biosciences, San José, Ca) per Standard Method 9215C (APHA 2012). Plates were inoculated using the spread plate method and then incubated for 7 days at 28 °C. Colonies were counted using a Quebec colony counter and results were recorded as CFU/100 mL.

### Statistical analysis

All statistics and graphs were conducted using RStudio v1.2.1335. An alpha value (*α*) of 0.05 was selected to determine the statistical significance (Dalgaard 2008). The normality of the data was determined using Shapiro–Wilk test. For non-parametric or non-normally distributed data (Shapiro–Wilk test, *p* < 0.05), Spearman’s rank correlation and Wilcoxon test were used to compare the BCYE agar count and Legiolert count for measured *L. pneumophila*. A linear correlation was conducted to develop a relationship between the culture method data and the Legiolert method data. In addition, the McNemar’s binomial analysis was used to compare these culture methods when used to determine only the presence or absence of *L. pneumophila* in the samples.

## RESULTS AND DISCUSSION

### Prevalence of *L. pneumophila*

A total of 185 water samples were analyzed for the presence of *L. pneumophila* using both BCYE agar and Legiolert culture methods, and the concentrations were reported in the log scale of measured concentrations (Table 1). Out of the 185 water samples, 174 were hot water samples with water temperature of 34 ± 1.6 °C (average ± standard deviation) from water heater, shower head, and four faucets; 11 were cold water samples with water temperature of 21 ± 0.4 °C that were taken only from faucet 3. Cold water samples showed higher free chlorine residuals with a range of 0.1–0.9 mg/L, whereas <0.1 mg/L was measured in most hot water samples (172/174, 99%). As expected, only 3 of 11 cold samples (27%) were positive and an average concentration of *L. pneumophila* in those positive samples was over 2 orders of magnitude lower than hot water samples (Table 1).

**Figure 1 |:**
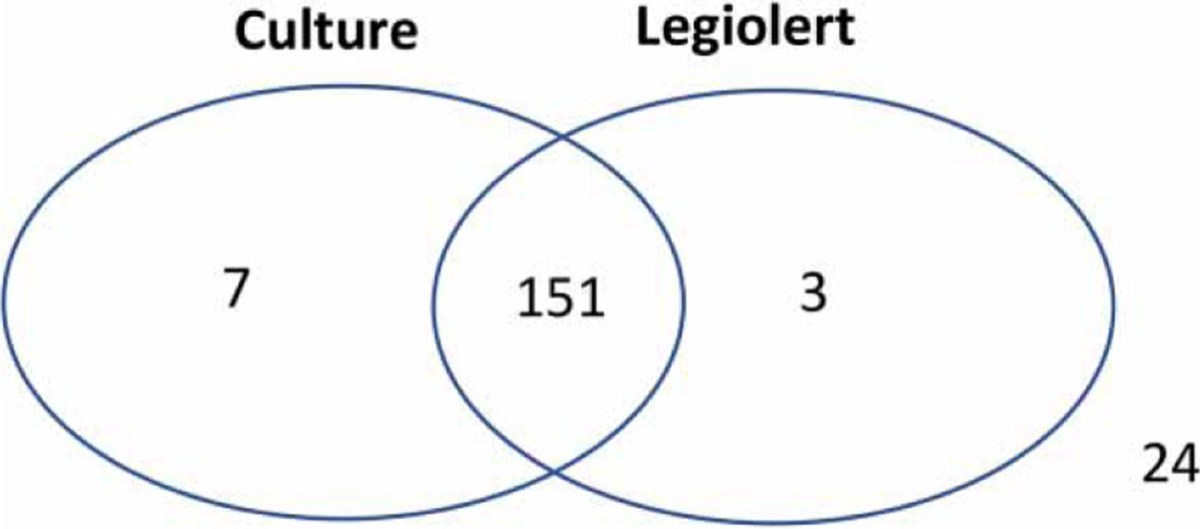
Venn diagram for the prevalence of *Legionella pneumophila* determined by two methods. Numbers outside the circles represent the numbers of samples that tested negative with both assays.

Of all the samples, 158 samples (85%) were positive determined by the BCYE culture method, compared with 154 samples (83%) using Legiolert (Figure 1). By combining the results from the two methods, the detection level slightly increased to 87% (Figure 1). While the vast majority of samples (82%, 151/185) were positive with both methods, some samples tested positive by one method; three samples tested positive using the BCYE culture but negative with Legiolert, and seven samples were positive with Legiolert but negative with the BCYE culture method (Figure 1). The McNemar’s statistics showed no statistical difference ( *p* > 0.1), indicating that both methods were equally sensitive for determining the prevalence of *L. pneumophila* in potable water. While this finding agrees with that reported by Petrisek & Hall (2018) for both potable and non-potable water, Spies *et al.* (2018) reported that the number of *L. pneumophila* positive samples with 100 mL determined by Legiolert were significantly greater than those by the BCYE culture method. In the latter study, six participating laboratories performed additional tests with 1 mL of samples independently, but there was no statistical difference in the prevalence of *L. pneumophila* by both methods. It was hypothesized that Legiolert would be less sensitive for *L. pneumophila* with higher sample volume analyzed due to greater levels of potential inhibitors from environmental water. Relatively high prevalence of *L. pneumophila* with 100 mL determined by Legiolert is a promising result in terms of practical application as well as regulation purpose (i.e., a sample analyzing volume of 10–100 mL). However, that study tested limited types of water and focused on municipal drinking water, suggesting further study on various types of environmental water to validate its use in field applications with a high volume of water.

Bacteria including *Legionella* in engineered water systems are known to exist in viable but non-culturable (VBNC) state, although there is controversy about the state of VBNC bacteria (Kirschner 2016). The existing standard culture methods may not detect cells that are viable, proliferating, and virulent, but non-culturable after stress (e.g., starvation, chemical disinfectants including chlorine, UV disinfection, heat treatment, etc.) (Ducret *et al.* 2014). Indeed, chlorine residual in premise plumbing water systems decreases the culturability of *L. pneumophila* (Alleron *et al.* 2008; Turetgen 2008). Alternative methods (e.g., cultivation-based and independent methods) to detect VBNC *L. pneumophila* are needed. Unfortunately, determining VBNC cells was beyond our original research scope. This further study makes the paper much stronger, particularly in public health risk assessment for *Legionella*.

### Comparison of quantification of *L. pneumophila*

**Figure 2 |:**
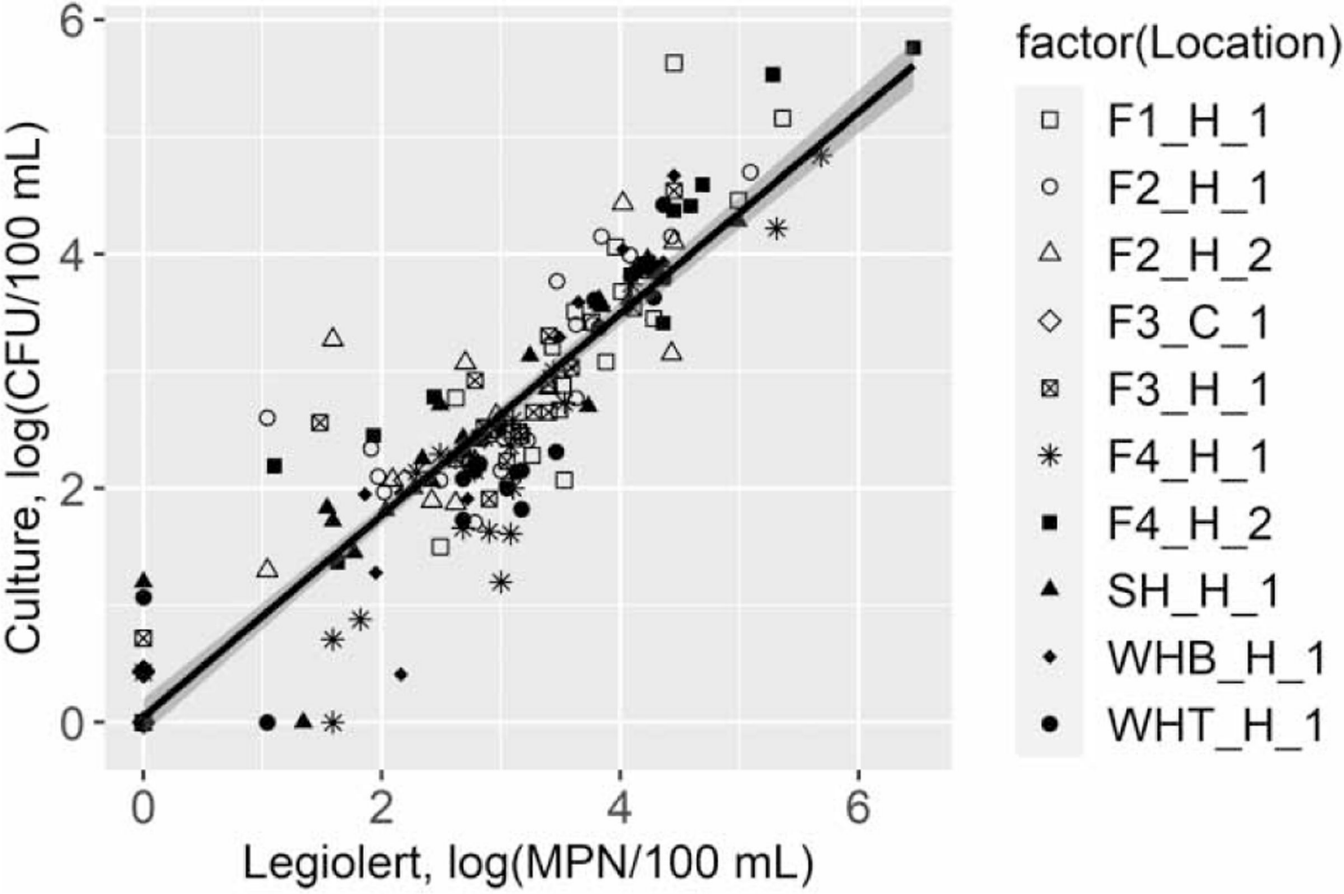
Correlation between the Legiolert method and the *Legionella* culture method of detected *Legionella pneumophila* in a simulated home plumbing system (*N* = 185, Spearman’s *ρ* = 0.91, *p* < 0.001). The values of zero represented below detection limit of *Legionella pneumophila* in water samples. The shaded area indicated the linear regression line (logCulture = 0.86 * logLegiolert) with 95% confidence intervals.

**Figure 3 |:**
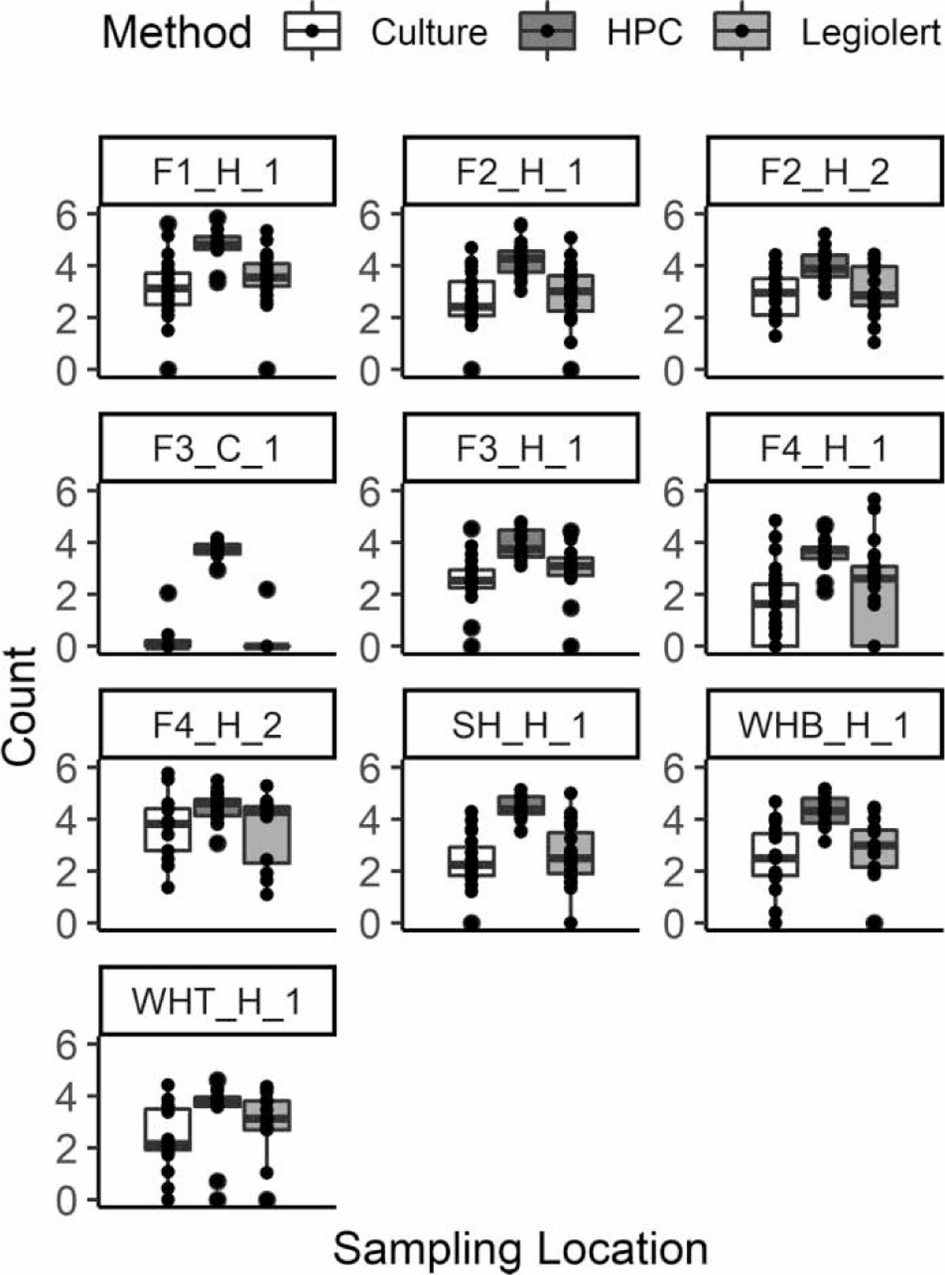
Boxplots for *Legionella pneumophila* by the culture and Legiolert method, and for heterotrophic plate count (HPC). Note that count for Legiolert: log (MPN/100 mL), Culture: log (CFU/100 mL), and HPC: log (CFU/mL).

Two culture methods showed a strong correlation with a statistical difference (Spearman’s *ρ* = 0.91, *p* < 0.001), and the Legiolert detection was slightly higher as indicated by a linear regression coefficient of 0.86 (Wilcoxon test, *p* = 0.005; Figure 2). In addition, HPC bacteria were detected in all the samples analyzed, and their concentrations were over 3 orders of magnitudes higher than *L. pneumophila* (Figure 3). It should be noted that HPC in cold water samples (21 ± 0.4 °C) were commonly detected at relatively high levels unlike *L. pneumophila* which were mostly undetectable. This observation was consistent with a previous study that there was no necessary correlation between HPC bacteria and *Legionella* positivity (Pierre *et al.* 2019). However, for those samples with positive *L. pneumophila* detection, the *L. pneumophila* levels were moderately correlated with HPC (Spearman’s *ρ* = 0.5, *p* < 0.001). This phenomenon was also observed by Ghanizadeh *et al.* (2016) where the presence of HPC bacteria detected in water from a hospital was associated with the amplification and occurrence of *L. pneumophila*. The possible explanation on the moderate correlation between HPC and *Legionella* in hot water is that these bacteria were colonized together in relatively mild environment of the HPS (i.e., low chlorine residuals in hot water). In contrast, there was no significant correlation in cold water where chlorine levels were relatively high (i.e., harsh condition against *Legionella*).

**Figure 4 |:**
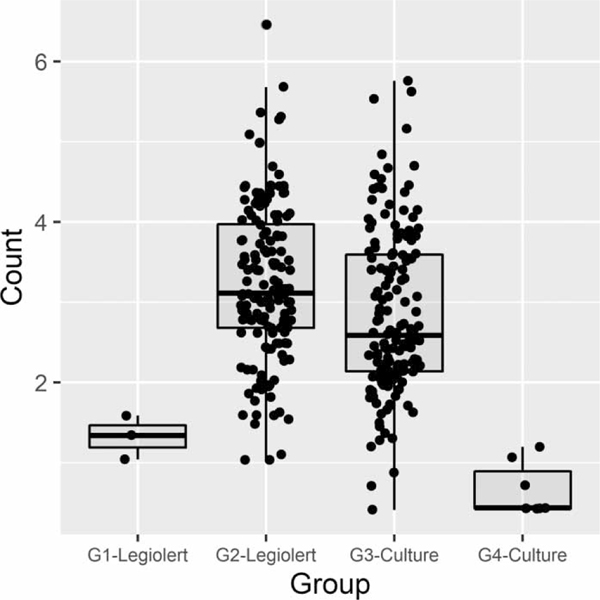
Boxplots of *Legionella pneumophila* with four group samples. G1 – Legiolert (Legiolert + and Culture —) (*N* = 3), G2 – Legiolert (Legiolert + and Culture +) (*N* = 152), G3 – Culture (Legiolert + and Culture +) (*N* = 152), and G4 – Culture (Legiolert — and Culture +) (*N* = 7). Count in *y*-axis: log (MPN/100 mL) for Legiolert or log (CFU/100 mL) for Culture.

Based on positives by Culture and/or Legiolert methods, all the water samples were grouped; G1 – Legiolert (Legiolert + and Culture —), G2 – Legiolert (Legiolert + and Culture +), G3 – Culture (Legiolert + and Culture +), and G4 – Culture (Legiolert — and Culture +). The concentrations of *L. pneumophila* for each group were presented in box plots (Figure 4). The groups G2 and G3 positive by both methods showed greater average concentrations than the other groups positive by one method. Some samples (*n* = 15) resulted in failing to identify *L. pneumophila* by the BCYE culture method due to overgrowth of non-*Legionella* bacteria on agar plates. In the present study, we intended to exclude these incomplete data sets for the grouping analysis. Interestingly, the relatively high concentrations with an average of 3.79 log (MPN/100 mL) were measured by Legiolert against the same samples. This result has a great deal of promise with regard to Legiolert’s applicability in environmental water particularly with high levels of non-*Legionella* bacteria. Further study on the inhibition of various types of water matrices with different levels of non-target bacteria is needed.

Besides environmental inhibitors, an incubation temperature is the other critical factor that impacts the culturability and specificity of *L. pneumophila*. There are the different incubation temperatures applied for the BCYE culture and Legiolert methods (i.e., 35 and 39 °C, respectively). It is well documented that *Legionella non-pneumophila* spp. prefer lower temperature for growth than *L. pneumophila* (Pryor *et al.* 2004; Costa *et al.* 2005; Wullings & van der Kooij 2006). More recently, Veenendaal *et al.* (2017) has reported that the elevated incubation temperature and BCYE media pH (40 °C and pH 7.3) can suppress the growth of *non-pneumophila* species, but not of *pneumophila*, demonstrating the improvement of the specificity of the standard BCYE agar method against *L. pneumophila*. Nonetheless, it is not feasible to acquire the complete selectivity for *L. pneumophila* without reducing its recovery rate while suppressing the growth of *non-pneumophila* species.

### Molecular confirmatory test for Legiolert

For each sample, three levels of dilutions (e.g., 1-, 10-, and 100-fold diluted samples) were analyzed. It was hypothesized that concentrations of *L. pneumophila* in positive wells after 7-day incubation are several orders of magnitude higher than the detection limit of qPCR assay. Interestingly, most undiluted liquid samples showed several orders of magnitude lower gene copies than 10-fold diluted samples (data not shown), indicating significant PCR inhibition. Moreover, few undiluted samples from Legiolert positive wells did not have *L. pneumophila* qPCR signals, but relatively high numbers of gene copies were determined in two dilutions (e.g., 10- and 100-fold dilutions) of the same samples. The 100-fold diluted samples were approximately 10 times lower quantities than 10-fold ones with over 10^6^ gene copies/well. Most Legiolert end users might not extract the well contents for either qPCR confirmation or obtaining isolates. For further molecular confirmatory tests, however, we suggested 10-fold diluted samples as an optimum dilution.

The qPCR assay confirmed the results of 245 out of 254 positive wells and 82 out of 82 negative wells, resulting in a high specificity of 96.5% (i.e., 3.5% false positives and 0% false negatives). Particularly, all 9 false positive samples were taken from small wells. Relatively high specificity of Legiolert is comparable to other published studies (Sartory *et al.* 2017; Petrisek & Hall 2018; Spies *et al.* 2018; Inoue *et al.* 2020; Scaturro *et al.* 2020). It should be noted that those studies performed secondary confirmations by sub-culture. Only one study by Inoue *et al.* (2020) used additional molecular confirmatory assays (e.g., sequencing and qPCR) when culture confirmation was not successful. In the present study, there was no evidence of interference by non-target microorganisms when using the Legiolert method. These samples, however, were collected from one premise plumbing water system. More recently, two studies have reported that non-*Legionella* bacteria derived from Legiolert positive wells (i.e., false positives) were identified as *Breundimonas* spp., *Ochrobactrum* spp., *Sphingomonas koreensis*, and *Elizabethkingia anophelis* (LeChevallier 2019; Buse *et al.* 2020). Moreover, in our preliminary study, on further evaluation of specificity against other non-*Legionella* environmental isolates from Legiolert positive wells, it appears *Pseudomonas aeruginosa* may give a false positive result (unpublished). Thus, there is a need for a more rigorous evaluation of specificity with various non-*Legionella* bacteria. In addition, further studies are needed to validate use of this product in more diverse sources of environmental water.

## CONCLUSIONS

To conclude, the Legiolert method performed with comparable efficacy to the traditional agar-based method in both the qualitative and quantitative detection of *L. pneumophila* in premise plumbing water samples. With the Legiolert method, however, quantitative results can be easily obtained after 7 days from the date of sample processing. This could allow many more water systems, utilities, and building operators to monitor their water for the presence of *L. pneumophila*. Moreover, according to the manufacturer, the Legiolert method allows for a much easier detection of *L. pneumophila* in water without the need for further confirmation. Indeed, our molecular confirmatory test demonstrated the relatively high specificity of Legiolert. However, the present study was limited by the testing water matrix and it identified non-*Legionella* isolates from positive wells. Thus, there is a need for further research. Studies which use more diverse water sources, as well as a further molecular confirmatory evaluation to investigate the characterization of non-*Legionella* isolates from false positive wells, would allow for greater confidence in the use of this product to detect *L. pneumophila* in environmental samples.

## Supplementary Material

Supplemental material

## Figures and Tables

**Table 1 |:** *Legionella* culture counts, Legiolert method counts, and heterotrophic plate counts (HPC) based on sampling sites and water conditions

				Culture, log (CFU/100 mL)		Legiolert, log (MPN/100 mL)		Culture/Legiolert		HPC, log (CFU/mL)		
							
Sampling location (Abbreviation)	Cold/Hot	1st/2nd Draw	Sample number	Avg	95% CI	Avg	95% CI	Positive count	Negative count	Sample number	Avg	95% CI

Faucet 1 (F1_H_1)	Hot	1st	20	3.13	0.55	3.55	0.48	19/19	1/1	17	4.98	0.36
Faucet 2 (F2_H_1)	Hot	1st	23	2.60	0.54	2.85	0.57	21/21	2/2	23	4.24	0.24
Faucet 2 (F2_H_2)	Hot	2nd	14	2.88	0.49	3.04	0.56	14/14	0/0	12	4.00	0.38
Faucet 3 (F3_H_1)	Hot	1st	20	2.45	0.50	2.73	0.59	18/17	2/3	18	3.94	0.28
Faucet 3 (F3_C_1)	Cold	1st	11	0.27	0.37	0.20	0.39	3/1	8/10	11	3.68	0.23
Faucet 4 (F4_H_1)	Hot	1st	31	1.48	0.49	2.12	0.59	21/21	10/10	33	3.67	0.19
Faucet 4 (F4_H_2)	Hot	2nd	13	3.72	0.69	3.81	0.85	13/13	0/0	23	4.48	0.22
Shower Head (SH_H_1)	Hot	1st	19	2.39	0.46	2.64	0.54	18/18	1/1	18	4.53	0.29
Water Heater Top (WHT_H_1)	Hot	1st	19	2.38	0.53	2.91	0.58	18/17	1/2	17	3.32	0.66
Water Heater Bottom (WHB_H_1)	Hot	1st	15	2.43	0.59	2.83	0.51	14/14	1/1	14	4.34	0.29

## HIGHLIGHTS

- *Legionella* spp. can be difficult to culture out of water systems and to recover from environmental sources.
- This study compared the culturability of *L. pneumophila* occurring in environmental water using the traditional agar-based method and the Legiolert product.
- The Legiolert method performed with comparable efficacy to the BCYE agar method in both the qualitative and quantitative detection of *L. pneumophila*.
