# Supplementary material for: Comparison of two culture methods for the enumeration of Legionella pneumophila from potable water samples
Source: J Water Health. Author manuscript; Available in PMC 2022 Jun 1. (PMC8358784; doi:10.2166/wh.2021.051)
Supplement: Supplemental material [file NIHMS1724938-supplement-Supplemental_material.docx]

**Supplemental material**


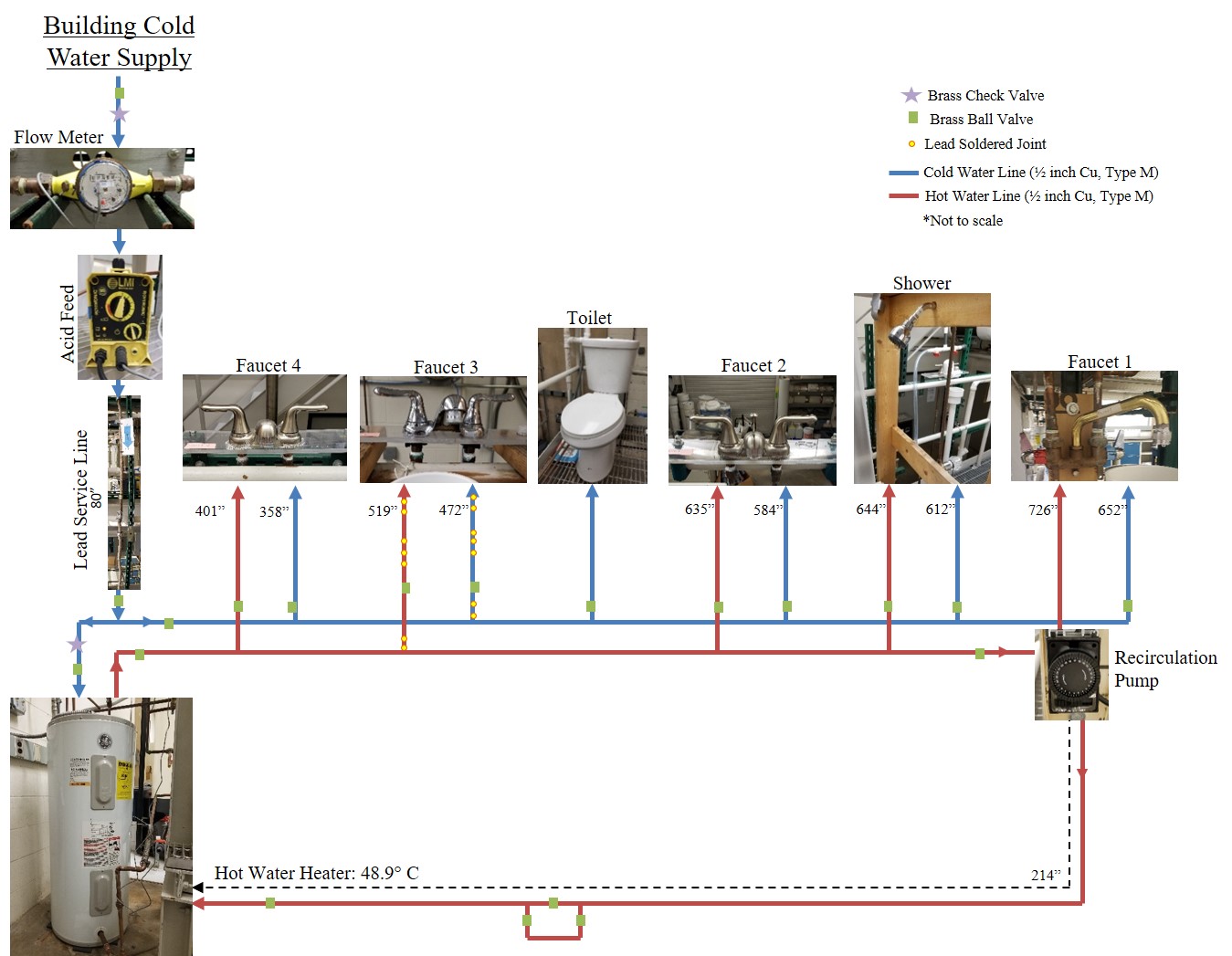


**Figure S1**. Schematic diagram of the premise plumbing model home. The distance from the point of water entry to the faucets (sampling points) for hot and cold water is presented (next to the pipeline).
